# Total Water, Phosphorus Relaxation and Inter-Atomic Organic to Inorganic Interface Are New Determinants of Trabecular Bone Integrity

**DOI:** 10.1371/journal.pone.0083478

**Published:** 2013-12-30

**Authors:** Ratan Kumar Rai, Tarun Barbhuyan, Chandan Singh, Monika Mittal, Mohd. Parvez Khan, Neeraj Sinha, Naibedya Chattopadhyay

**Affiliations:** 1 Centre of Biomedical Research, SGPGIMS Campus, Lucknow, India; 2 Division of Endocrinology and Center for Research on Anabolic Skeletal Targets in Health and Illness (ASTHI), CSIR-Central Drug Research Institute, Jankipuram Extension, Lucknow, India; University of Pittsburgh School of Medicine, United States of America

## Abstract

Bone is the living composite biomaterial having unique structural property. Presently, there is a considerable gap in our understanding of bone structure and composition in the native state, particularly with respect to the trabecular bone, which is metabolically more active than cortical bones, and is readily lost in post-menopausal osteoporosis. We used solid-state nuclear magnetic resonance (NMR) to compare trabecular bone structure and composition in the native state between normal, bone loss and bone restoration conditions in rat. Trabecular osteopenia was induced by lactation as well as prolonged estrogen deficiency (bilateral ovariectomy, Ovx). Ovx rats with established osteopenia were administered with PTH (parathyroid hormone, trabecular restoration group), and restoration was allowed to become comparable to sham Ovx (control) group using bone mineral density (BMD) and µCT determinants. We used a technique combining ^1^H NMR spectroscopy with ^31^P and ^13^C to measure various NMR parameters described below. Our results revealed that trabecular bones had diminished total water content, inorganic phosphorus NMR relaxation time (T_1_) and space between the collagen and inorganic phosphorus in the osteopenic groups compared to control, and these changes were significantly reversed in the bone restoration group. Remarkably, bound water was decreased in both osteopenic and bone restoration groups compared to control. Total water and T_1_ correlated strongly with trabecular bone density, volume, thickness, connectivity, spacing and resistance to compression. Bound water did not correlate with any of the microarchitectural and compression parameters. We conclude that total water, T_1_ and atomic space between the crystal and organic surface are altered in the trabecular bones of osteopenic rats, and PTH reverses these parameters. Furthermore, from these data, it appears that total water and T_1_ could serve as trabecular surrogates of micro-architecture and compression strength.

## Introduction

Skeleton maintains its unique biomaterial composition and strength by the dynamic process of remodeling involving bone resorption by osteoclasts and bone formation by osteoblasts. Cortical bones, accounting for 80% of the weight of a skeleton primarily afford bone biomechanical strength. Trabecular bones, accounting for the remaining 20% of the weight of the skeleton serve to maintain mechanical strength and more importantly, control mineral (calcium and phosphorus) metabolism and are metabolically more active than the cortical bones[1], [2]. Loss of trabecular bones may occur as a result of increased remodeling rate and/or a negative remodeling balance and is a hallmark of postmenopausal osteopenia[3]. Pregnancy and lactation are two physiologic states, when mobilization of calcium from bone to blood and milk necessitate bone loss to occur, mostly at the trabecular sites[4]. Restoration of the lost bone is mostly complete postpartum/post-weaning [5], [6]. However, bone restoration or new bone formation is only achieved by administering parathyroid hormone to postmenopausal women suffering from osteoporosis [7].

The composite biomaterial of bone is comprised of collagen, water and substituted apatite. Organic part consists predominantly of type 1 collagen (90%), inorganic part mostly consists of carbonated hydroxyapatite (HAP) and water accounts for 10–12 wt% of bone[8]. The unique ultra-structural arrangements and interaction with organic component, inorganic mineral and water confer strength and elasticity properties to the bone. An ordered structural water layer was found between the inorganic mineral structures and the organic (collagen) part which appeared to serve important functions pertaining to the bone biomechanical property[9].

To advance the clinical assessment of fracture risk, nuclear magnetic resonance (NMR) is being developed to quantify water in the bone. There are two kinds of water reported in the literature; a) free, residing in the Harversian and lacuna-canalicular system and b) bound, associated with inorganic components and collagens[10], [11], [12]. Various studies which so far have been performed to understand the role of water in bone revealed the following: i) mechanical properties of bone was reduced by dehydration of bone matrix[13], ii) more displacement of water to osteoid occurred with the mineralization[14], iii) decrease in bound but not free water content of bone due to aging[15], iv) hydration influencing the mobility of amino acid residues in collagen, specifically hydroxyproline Cγ, located on the periphery of triple helix and fibril[16], v) presence of hydrogen bonding network between collagen and surrounding environment through water molecules[17] and vi) shorter distance between collagen and inorganic surfaces with loss of water[9], [18], vii) dehydration reduces the mobility of collagen amino acid residues in cartilage and rehydration restores structure and mobility[19]. Presently, there are no systematic studies investigating the role of water in trabecular bones of osteopenic and osteopenia recovery models taking into account the established parameters such as bone mineral density, microarchitecture and strength. We hypothesized that interaction of water with mineral and matrix of bone is altered in osteopenia, and which is reversed upon bone formation.

In the present study, using femur epiphysis (trabecular bone) of the following groups of rat - adult ovary intact (sham), osteopenic (ovariectomized, Ovx), lactating and Ovx+PTH, we studied natural organic and inorganic environment and their interaction with water. Osteopenic rats (induced by lactation or bilateral Ovx for six months) and restoration of trabecular bones (in Ovx+PTH) in comparison to sham were evaluated by bone mineral density (BMD), microarchitecture and bone strength. In these groups, we then performed 1D ^1^H NMR to measure total water content relative to OH bound to Ca^++^ surface and cross peak intensity in 2D ^1^H-^31^P HetCor to measure bound water content relative to inorganic content. Relaxation parameter (T_1_) of ^31^P was used to evaluate the integrity of inorganic mineral and ^13^C[^31^P] Rotational Echo Double Resonance (REDOR) allowed the determination of distance between collagen side chain residues and inorganic surface as well as the amount of water present between them. Finally, we performed a correlation analysis between the NMR parameters representing bone biomaterial integrity at the atomic scale with the established set of parameters, including BMD, micro-architecture and compressive strength that are commonly invoked in the preclinical situation to demonstrate structural integrity of trabecular bones, with the aim to investigate if the former parameters could serve as the surrogate of any of the later. Taken together, our study will help to elucidate a novel ultra-structure of bone.

## Results

### Assessment of trabecular bone quality by established parameters

Induction of trabecular osteopenia in adult rats by bilateral Ovx serves as the WHO-recommended preclinical model for postmenopausal osteoporosis. PTH is the only anabolic therapy that is known to restore bones that are lost at the trabecular sites due to Ovx. Pregnancy and lactation represent osteopenic states under physiological condition. However, no study is available comparing the extent of osteopenia between peak lactation and long-term estrogen deficiency by bilateral Ovx (six months).

**Figure 1:**
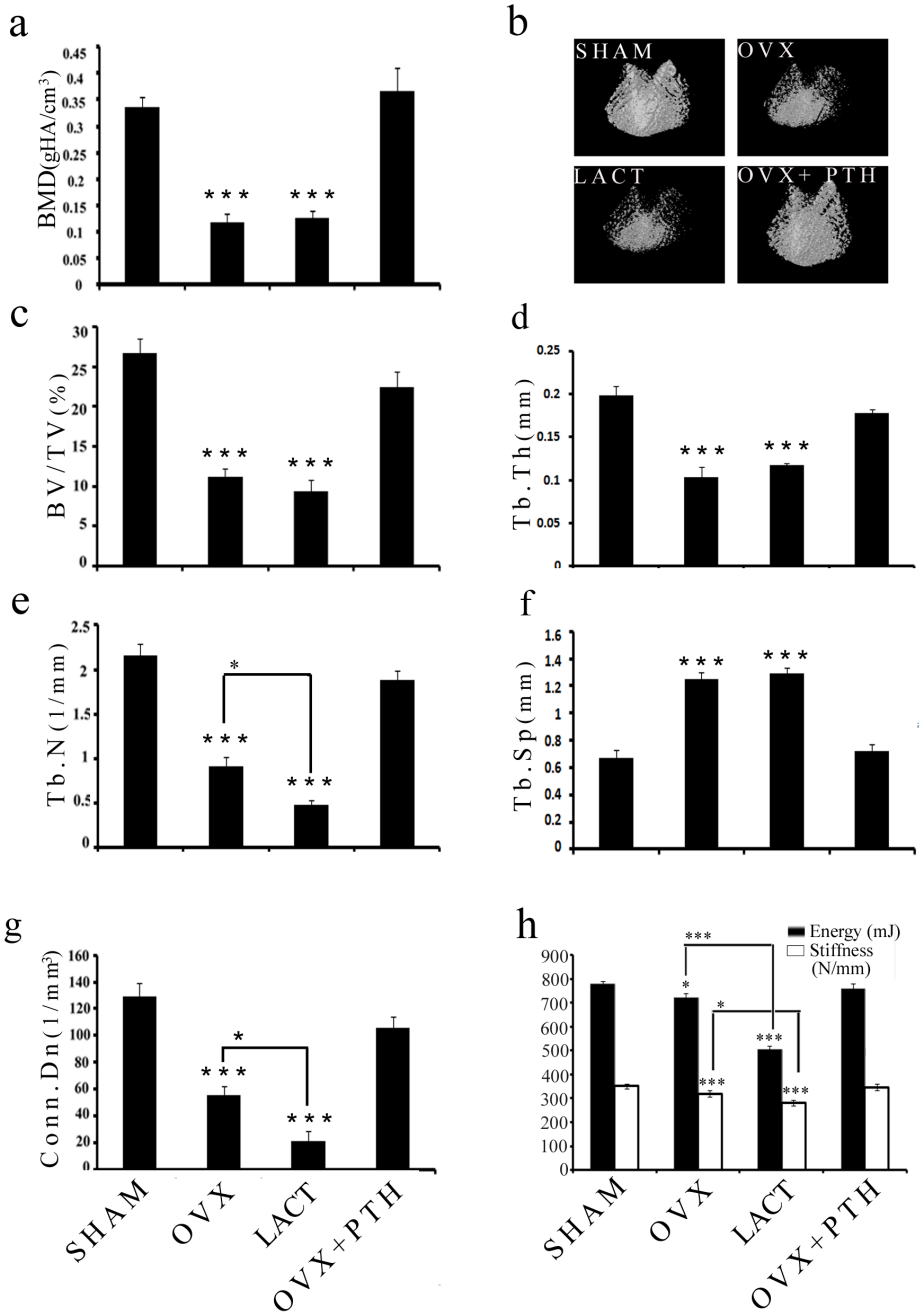
Induction of trabecular osteopenia and bone regeneration by PTH is shown in femur epiphysis of various groups of rats. (a) BMD; (b) representative µCT images of femur epiphysis of various experimental groups; (c–g) µCT analysis (3-D reconstruction) of various trabecular parameters, bone volume/trabecular volume (BV/TV), trabecular thickness (Tb.Th), trabecular number (Tb.N), trabecular spacing (Tb.sp), connectivity density (conn.D); and (h) compression test of femur epiphysis showing energy and stiffness.^*^ *P*<0.05, ^***^ *P*<0.001; data expressed as mean ± SEM (n = 6 rats/group).

We assessed femur epiphysis (comprising of trabecular bones) in various groups by BMD (**Fig 1a**), µCT (**Fig 1b–g**) and compressive strength (**Fig 1h**). Sham operated rats (ovaries intact) served as control for intact trabeculae. BMD, trabecular bone volume (BV/TV), Tb.Th, Tb.N and Conn.D were significantly reduced in Ovx and lactation groups compared to sham (**Fig 1a–g**). There were no differences in these parameters between the sham and Ovx+PTH groups. Tb.N and Conn.D were least in the lactating group compared with other groups. Tb.sp was increased in Ovx and lactation groups compared to control, whereas it was not different between the sham and Ovx+PTH groups (**Fig 1f**). Energy to compression (resistance to strain) was reduced in Ovx and lactation groups compared to sham or Ovx+PTH groups. Lactation group had significantly lower compression energy compared to Ovx. Similar results were obtained when stiffness was compared between various groups (**Fig 1h**).

### Assignment of ^1^H NMR spectra

**Figure 2:**
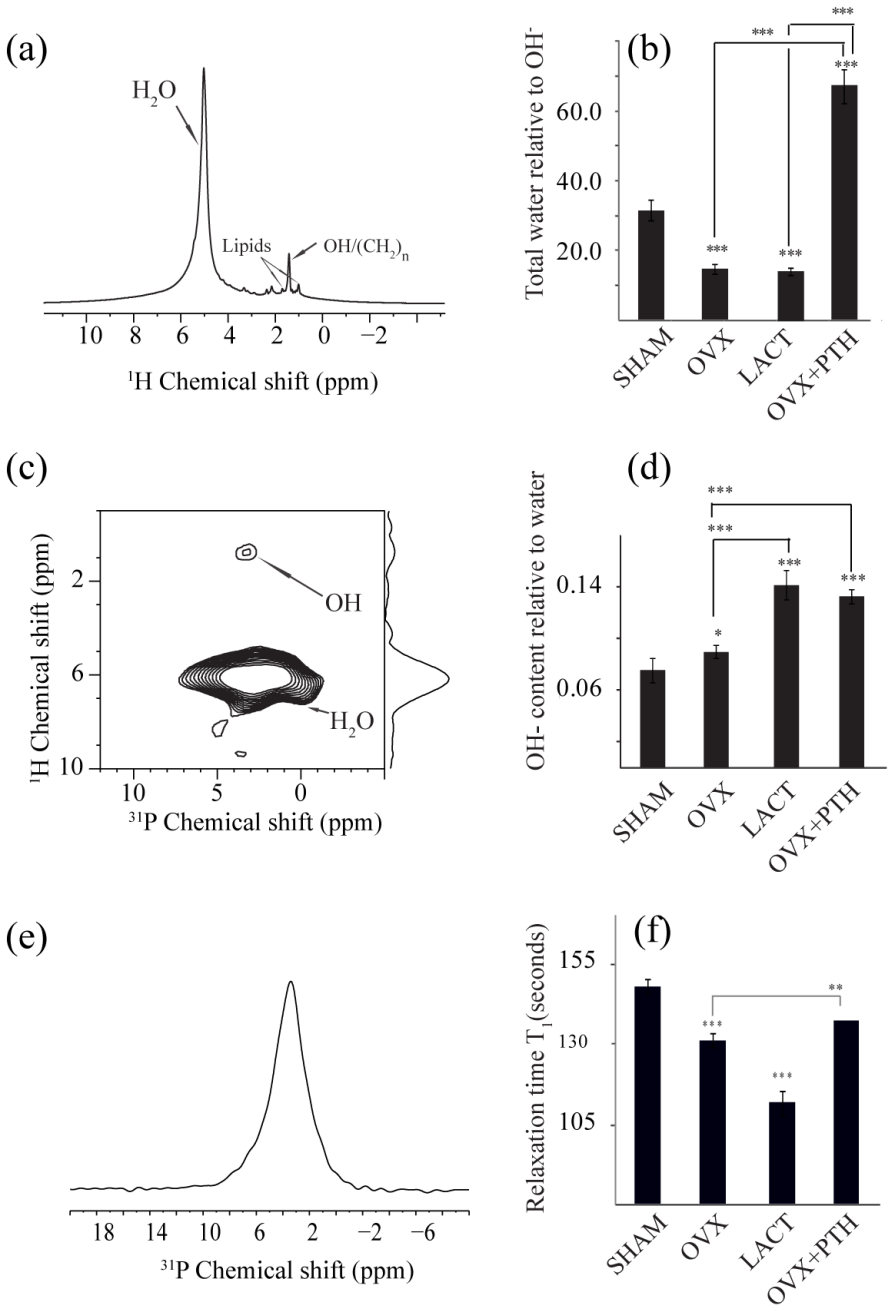
Various ssNMR parameters are shown in femur epiphysis of different groups of rats. (a) ^1^H MAS NMR spectra; (b) total water content relative to OH variations; (c) ^1^H-^31^P HeTCor NMR spectra; (d) bound water content relative to inorganic OH variations; (e) ^31^P spectra; and (f) bar plot of T_1_ values of ^31^P. ^*^ *P*<0.05,^**^ *P*<0.01 and^***^ *P*<0.001; data expressed as mean ± SEM (n = 6 rats/group). Representative spectra in (a), (c) and (e) are from SHAM group.

^1^H NMR spectra of femur epiphysis are shown in **figure 2a**. All peaks were externally referenced to ^1^H NMR spectra of water at 4.7 ppm. In all bone samples, there were six distinct peaks in ^1^H NMR spectra, out of which water (5.05 ppm) and hydroxide (OH, 1.4 ppm) peaks were predominant. These assignments were consistent with previously reported values [17], [20]. The peak at 1.05 ppm and 1.5 ppm were assigned as bone lipid proton [21] and the peak at 2.2 ppm was assigned to water occupying isolated OH vacancies. Finally a small peak at 2.3 ppm was assigned to the water molecule in hydroxide ion channel [21]. This peak was also observed in carbonated apatite. It should be noted that our bone samples were completely devoid of marrow, which is a rich source of cells. Hence, we do not observe most of the resonances corresponding to lipids and choline [22].

### Assessment of trabecular bone by NMR

In the sham rats, there was one sharp peak of water at 5.05 ppm. Water behavior in the remaining three groups was markedly different from the sham as these bones exhibited two peaks at 5.05 and 5.4 ppm (Figure S1). These two peaks appeared to represent bound and free water. Free water could reside in the pores (lacunar canaliculi) and bound water is mainly associated with collagen and inorganic surface. To determine the ratio of total water content with respect to OH, we carefully integrated water peak (5.05–5.4 ppm) with respect to OH resonance (1.4 ppm). With intact bones, Zhu et al observed very little variation in peak at 1.4 ppm after dehydration [17]. This finding suggested that peak at 1.4 ppm can serve as internal reference to assess relative change in water content. In the sham group, the total water/OH ratio was 31±7.5, which was higher than the osteopenic, Ovx+ vehicle (14.8±3.35) and lactating (14.2±2.64) groups. The ratio in Ovx+PTH group (the trabecular restoration model) was 67±12 (Figure 2b).

We next performed 2D ^1^H-^31^P heteronuclear correlation experiment (HeTCor) to gain insights into the inorganic arrangements in the close vicinity of ^31^P atoms. HeTCor has previously been utilized successfully to probe the atomic scale structural details [10], based on ^1^H-^31^P dipolar coupling. It selectively excites ^1^H signal associated with water and organic component of the bone, and thus provides spatial arrangements of nearby inorganic bone mineral. HeTCor spectra showed two well-resolved peaks at 0.4 ppm (OH) and 4.8 ppm (bound water) (**Figure 2c**). The bound water peak intensity depends on coupling with various ^1^H resonances present in bone. Because HeTCor exploits dipolar coupling between ^1^H and ^31^P nuclei, and is also dependent on the distance between coupled nuclei (1/r^3^), the ratio between the bound water content to OH, therefore, reveals OH content associated with bound water. This ratio thus serves to determine the amount of OH present nearby inorganic surface. Although cross-peak intensity of HetCor is not quantitative in nature mainly due to variation in cross-polarization transfer efficiency, yet it is possible to obtain information about bound water content. ^1^H to ^31^P cross-polarization (**Figure 2d**) showed that amongst various groups, the intensity of OH content relative to bound water in the femur epiphysis of sham group was the least and highest in the Ovx+PTH and lactating groups, whilst in the Ovx+vehicle group, the value was modestly but significantly higher than the sham.

We next assessed bone inorganic phosphorous (^31^P) by calculating ^31^P relaxation (T_1_). ^31^P T_1_ has previously been correlated with bone strength [23]. It is also reported that ^31^P T_1_ of apatite depends on structural OH^-^, which in turn influences mineral crystallinity. Consistent to this finding, non-crystalline synthetic apatite exhibited longer T_1_ than crystalline HAP [24]. Thus, ^31^P T_1_ appears to reflect changes in inorganic OH^-^ as well as alterations in the inorganic component of bone. We found 30% reduction in the T_1_ value in lactation group compared to sham (**Figure 2e,f**). T_1_ was also reduced in the Ovx group however; its extent was less than the lactation group. In the Ovx+PTH group, T_1_ was significantly greater than the Ovx group.

**Figure 3:**
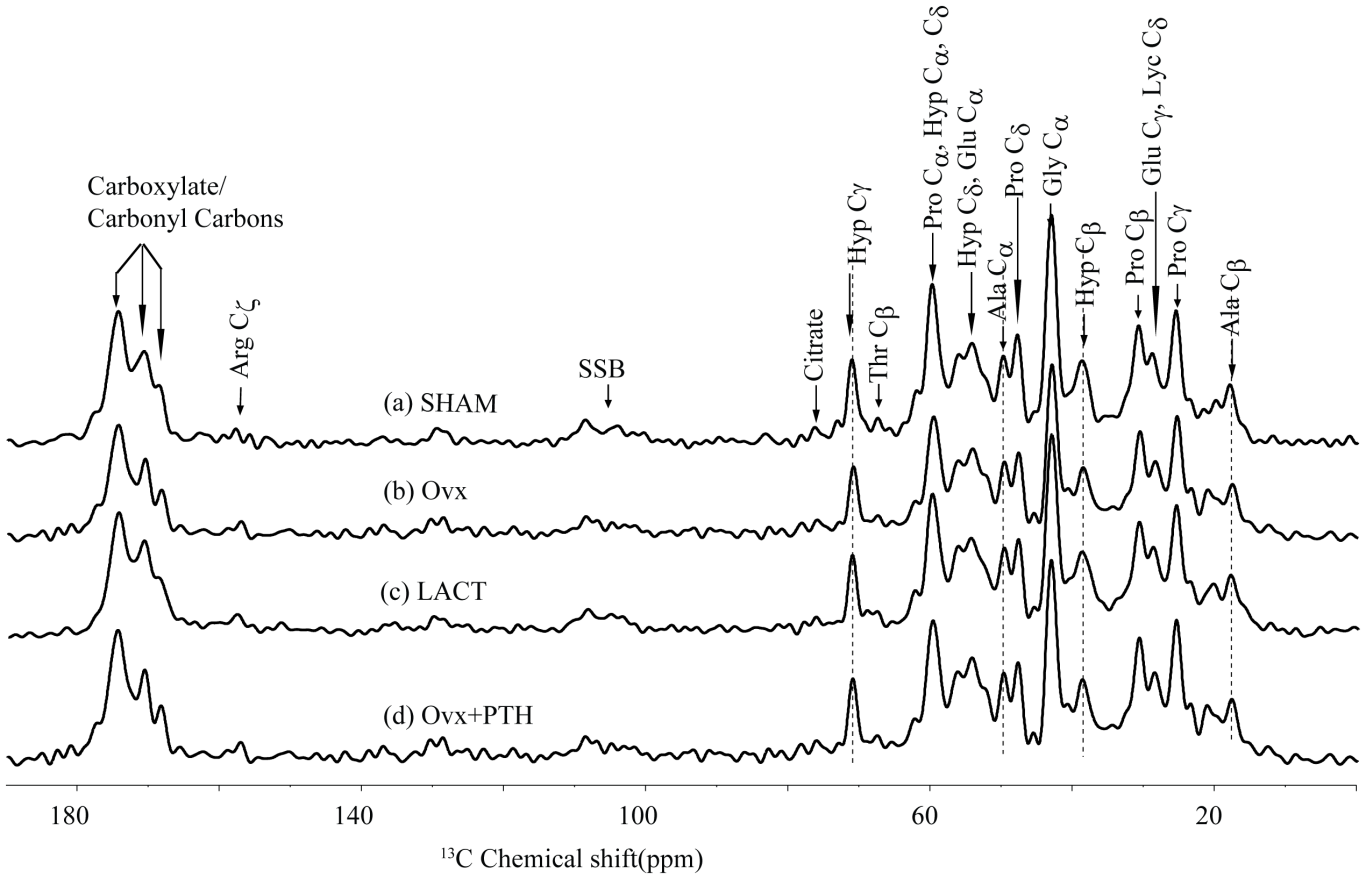
Representative ^13^C spectra of femur epiphysis of various groups of rat are shown.

We next assessed structural changes in the organic component of bone by ^13^C spectra as it principally represents resonance from type 1 collagen (**Figure 3**). Ala Cβ resonance at 17.6 ppm was similar to previous studies, which confirmed that collagen was in the triple helical state [25], [26]. Although, hydration-induced changes that are reported earlier suggest that as water is eliminated from bone matrix, citrate resonance at 76 ppm disappears and well resolved carbonyl peaks merge together [17], we, however, failed to observe these patterns of change in the present study. There was no change in collagen line-width (between 17 to 76 ppm) between the various groups. Given that isotropic chemical shifts are very sensitive markers for secondary structural changes in protein, intact collagen spectra between the different groups indicate unaltered triple helix arrangement of the collagen fibrils. In addition, carbonyl peaks were also unchanged between the groups.

### Determination of inter-atomic space between the inorganic and organic surface in trabecular bones by REDOR

**Figure 4:**
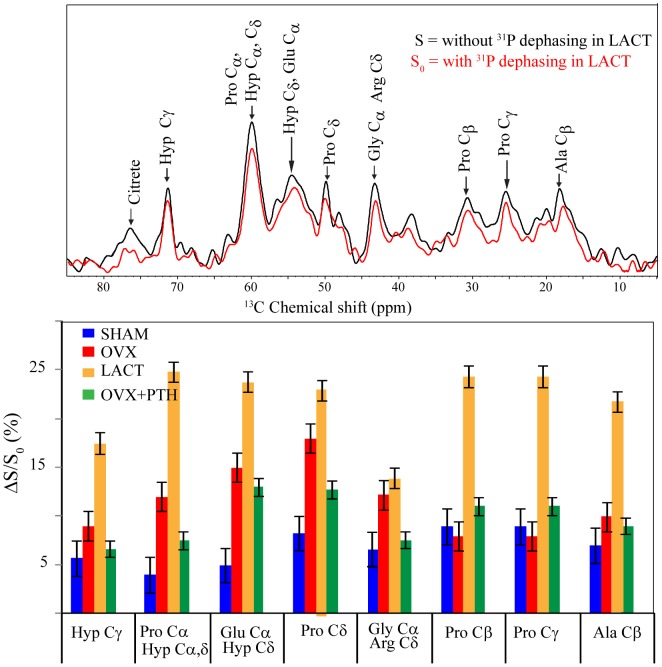
^13^C REDOR spectrum showing dephasing (top) and dephasing variations corresponding to different collagen resonances in femur epiphysis of different groups (bottom). Representative ^13^C NMR spectrum with assignment of lactation bone sample showing dephasing at 16 ms is shown at the top. The error bars in the dephasing of individual peaks were determined by error introduced due to signal to noise ratio. Error bar was calculated based on percentage contribution due to signal to noise ratio and summed together. See Results for details.

We performed ^13^C[^31^P] REDOR NMR experiment to measure inter-atomic distance between organic surface and ^31^P of inorganic components (HAP) in the trabecular bones of different groups within the precision limit of 0.1 Å. The amount of dephasing observed is inversely proportional to the distance between nuclei. The choice of REDOR dephasing period is critical to understanding the inorganic to organic interface. We have used 16 ms dephasing time to compare different interfaces in various samples. Earlier, we performed REDOR experiment on goat bone to study dehydration-induced structural changes between organic surface and HAP [9]. We found that 16 ms is appropriate time for recording significant changes in REDOR dephasing curve and ΔS (S-S_0_)/S_0_ allows distance measurement between ^13^C and ^31^P spin pairs. In the present study, using rat trabecular bone samples, we observed dephasing from almost all carbon signals of organic surface at 16 ms dephasing time (**Figure 4**). The signal at 76 ppm assigned as citrate dephased faster than any other carbon signal indicating it to be the closest to inorganic surface. The amount of dephasing corresponding to different residues of collagen side chain has been measured (**Figure 3**). The change in dephasing can be further expressed in terms of distance between collagen to inorganic surface. Maximum dephasing in Pro Cδ, Glu Cδ and Lyc Cδ in the lactating group was 24%. The sham group had a minimum dephasing. The distance between collagen side chains Hyp Cγ and inorganic surface was longest in the sham (9.7±0.7 Å) and shortest in lactating rats (8.3±0.7 Å). This distance was lesser in Ovx+ vehicle group (8.6±0.7 Å) compared to the sham but increased in the Ovx+PTH group (8.9±0.6 Å) compared to the two osteopenic groups.

### Correlation analysis between NMR and established parameters of trabecular integrity

**Figure 5:**
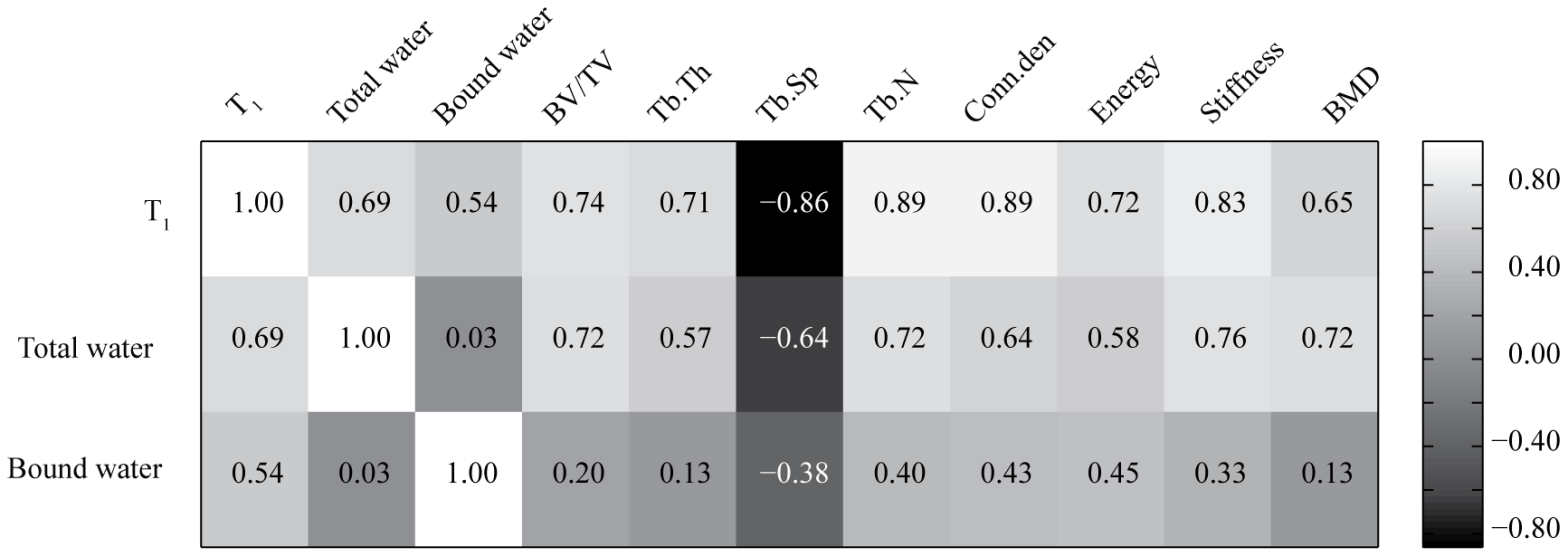
Correlation analysis between ssNMR parameters with established µCT parameters that are indicative of structural and functional integrity of trabecular bone.

**Figure 5** showed correlation of various NMR parameters with established trabecular parameters. T_1_ correlated highly with all the parameters but more strongly with Tb.N and conn.D (*r* ^2^ = 0.89 for both), Tb.sp (*r* ^2^ = −0.86) and stiffness (*r* ^2^ = 0.83). Total water also showed strong correlation with all the aforementioned parameters but weaker than T_1_. BMD, trabecular bone volume (BV/TV) and Tb.N (*r* ^2^ = 0.72 in all three cases) were best correlated with total water. Bound water did not exhibit strong correlation with any of the established trabecular parameters.

## Discussion

The aim of the present study was to understand the interaction of water with inorganic and organic components in the native state in the cancellous bone of osteopenic and osteopenia recovery models in adult rats. Two osteopenic models included Ovx and peak lactating rats whereas the recovery model was obtained by PTH treatment to Ovx rats after osteopenia was established [43]. Assessments of BMD, micro-architectural parameters and compressive strength (resistance to strain) of the femur epiphysis were made to determine the structural and functional integrity of trabecular bone. The extent of loss was compared between two osteopenic groups and following two salient observations were made; a) based on BMD, both groups were comparable but lower than the sham and b) micro-architectural deterioration was the worst and compressive strength was lowest in lactating rats. Overall, the severity of trabecular loss in the lactating rats was more than Ovx rats (devoid of E2) for six months. However, the former condition is expected to reverse in the natural course after weaning but the latter requires bone regenerative intervention, i.e. PTH. Indeed, all the trabecular parameters were comparable between Ovx+PTH and sham (ovary intact) suggesting complete restoration of architecture and strength by PTH.

Using femur epiphysis of these validated osteopenic and osteopenia restoration models, we set out to study NMR parameters. The following NMR observations were made in the osteopenic groups in comparison to the sham: i) total and bound water were both reduced in the osteopenic groups wherein reduction in total water was comparable between the groups but bound water was more diminished in the lactating group over the Ovx, ii) T_1_ was dramatically reduced in lactating followed by Ovx group and iii) the interface between the collagen and inorganic surface was decreased in both osteopenic groups, but it was least in the lactating group. PTH treatment to Ovx rats resulted in a greater increase in total water but decrease in bound water over the sham whereas T_1_ and inter-surface distance between collagen and inorganic surface were largely comparable between these two groups. Thus restoration of trabecular bones by PTH was associated with restoration of the majority of the altered NMR parameters in osteopenic rats.

REDOR is an extremely sensitive method as it offers atomic-scale resolution of the inorganic and organic interface of bone. As shown in **Figure 3**, various amino acid signals in collagen of different groups exhibited a unique dephasing pattern, suggesting that organic and inorganic interface between the bone loss and restoration models was significantly different. This change in dephasing appears to reflect the cross-linking between the interface and hence the ultra-structure. Aged/diseased collagen has higher cross-links than fresh collagen (18). Interface distance was smallest in lactating rats compared to other groups. This change in interface distance appeared to be associated with free water in bone, which also decreased in osteopenic bones. This finding attested to earlier studies showing that decrease in interface distance was associated with water loss in bone as well as in osteoporotic bones (8). Corresponding ^1^H MAS spectra of different rat bones showed a distinct water behavior (**Figure 2a**). Water signal in ^1^H NMR spectra was sharp in sham whilst relatively wider and blunted in the osteopenic as well as Ovx+PTH groups. This difference might be due to the loss in organized bone water. Ovx+PTH group exhibited a robust increase in free water that was greater than the sham, but the change in dephasing was marginally lower than Ovx; suggesting that PTH treatment to osteopenic rats partially recovered the interface distance.

Similar to the REDOR, ^31^P relaxation parameter (T_1_) of inorganic surface was altered (**Figure 2e**) evident from its decreased values in the Ovx and lactating groups compared to sham but not in Ovx+PTH group. It is surmised that the difference in T_1_ was due to a more organized inorganic surface in the sham than in Ovx or lactating rats. In addition, when the crystallinity of the inorganic surface gets disrupted, water molecules relax faster via ^1^H-^31^P dipolar coupling. It has been reported earlier that dehydration disrupts collagen to inorganic interface (8). Data from our REDOR experiments indicate greater proximity between the inorganic surface and collagen in the osteopenic groups compared to sham, which could be attributed to loss of interface water in these groups. In Ovx+PTH group, REDOR data show a significant increase in overall space between the collagen side chain residues and inorganic surface over the osteopenic groups. These data offer a novel structural insight into rebuilding of trabecular bones by PTH, i.e. micro-architectural restoration induced by the hormone also tends to establish spatial arrangements of organic to inorganic surface similar to that of the sham group at the atomic (Ångström) level. Interestingly, total water increased but bound water decreased significantly in the Ovx+PTH group compared to the sham. Increased total water could be contributed by the increase in blood flow due to angiogenesis that occurs during new bone formation [27], [28]. Whether the greater increase in total water in the Ovx+PTH group compensated for decreased bound water as well as contributed to increase in the distance between collagen and inorganic interface remain to be investigated. It is noteworthy that the extent of reduction in bound water in the Ovx+PTH group was comparable to lactating rats (physiological osteopenia that is normally reversed after weaning), suggesting that greater loss in bound water could be a precondition for reversal of osteopenia. Recent data suggest that osteocytes in addition to osteoclasts contribute to the demineralization of skeleton during lactation by remodeling perilacunar/canalicular matrix, by a process called osteocytic osteolysis [29]. PTH-related protein (PTHrP), whose levels are elevated during lactation, interacts with PTH receptor 1, the same receptor via which PTH signals in osteoblasts and osteocytes. Indeed, osteocytic osteolysis has also been reported in response to exogenous PTH [30], [31]. It is possible that the demineralization of canalicular matrix during lactation and intra-trabecular tunneling induced by PTH to increase Tb.N (discussed below) involve osteocytic osteolysis resulting in loss of bound water in the lacuna-canalicular network.

Amongst the various NMR metrics, T_1_ showed strongest correlation with the micro-architectural parameters (particularly Tb.N, conn.D, Tb.sp, BV/TV and Tb.Th) followed by the strength indices. Tb.Th and Tb.sp are key measures characterizing the 3D structure of trabecular bone. Thus, T_1_ appears to closely represent the 3D trabecular network and could serve as its surrogate. Because Tb.sp represents non-bone part and is inverse of the mean distance between trabeculae, a more negative value to this parameter therefore strongly correlated with T_1_. Total water had the strongest correlation with stiffness followed by BMD, BV/TV and Tb.N. Bound water did not show strong enough correlation with any of the micro-architecture and strength parameters as well as BMD. Bound water data showed a discrepancy as it was decreased more in lactation as well as Ovx+PTH groups over the Ovx group. Greater loss of bound water in the lactation over Ovx correlates with greater induction of osteopenia in the former over the latter. However, its decrease in PTH treatment (bone gain) being comparable to lactation (the most severe bone loss), for which a plausible explanation has been presented in the preceding paragraph, might have contributed to the lack of correlation of this ssNMR parameter with the established parameters of trabecular integrity and strength. Trabecular bone loss begins with the erosion of individual trabecular surfaces by osteoclasts thereby reducing Tb.Th and subsequently Tb.N and connectivity (conn.D). Both osteopenic groups showed loss in BMD and deterioration of micro-architectural indices however, the lactating group had the lowest Tb.N and conn.D amongst the osteopenic groups and so was its T_1_ value. PTH is known to increase BV/TV primarily by induction of Tb.N and connectivity [32]. Initially, PTH stimulates new bone formation in the existing trabecular surfaces to increase thickness that is associated with increased vascularization of the trabeculae, which could explain a robust increase in total water in Ovx+PTH group [33]. Subsequently, the tunneling process contributed by osteoclasts and/or osteocytes splits a thickened trabacula longitudinally into two thinner trabaculae by bone remodeling thereby increasing Tb.N and creating a more extensive and better connected trabecular network to reduce Tb.sp by PTH treatment [34], [35]. Formation of trabecular bones and restoration of micro-architecture by PTH is known to result in superior biomechanical competence. Strong correlation of T_1_ with BMD and micro-architectural and strength parameters suggest that it could serve as a surrogate for all of the above, particularly number, volume, connectivity and spacing of trabeculae and stiffness. Although these initial predictive results are compelling, they are based only on simple univariate regressions, and more complex multivariate prediction models could potentially provide further accurate predictions.

Our studies have the following limitations: a) NMR parameters of lactating rats post-weaning as a model of physiological restoration were not studied, b) correlation of total water and T_1_ with biochemical markers of bone loss (urinary or serum collagen type 1 cross-linked C-telopeptide) or gain (serum type 1 procollagen, C-terminal/N-terminal) were not performed and c) due to rather extended signal averaging time associated with REDOR measurement, analysis of multiple samples by this technique in each group which is required for statistical analysis, could not be performed. The paramagnetic relaxation properties of copper (II) can be used to reduce the REDOR experimental time as suggested earlier by Marque et al., in which ^13^C spectra of collagen recorded without reducing the spectral resolution and thus enabling faster data acquisition[36].

In conclusion, our study demonstrated that total water, phosphorus (a key element of the bony crystal) relaxation and atomic space between the crystal and organic surface are altered in the trabecular bones of osteopenic rats and PTH reverses these parameters. This study also implies that T_1_ and total water could be translated in future towards developing novel non-interventional structural and composition markers of bone using magnetic resonance imaging technology for improved management of post-menopausal osteoporosis.

## Materials and Methods

### Animals and experimental design

The animal experimental protocol in this study was approved by the Institutional Animal Ethical Committee (IEAC) at CSIR-Central Drug Research Institute (CDRI) and the study was conducted in compliance with the standards and guideline mentioned by the Committee for the Purpose of Control and Supervision on Experiments on Animals (CPCSEA). The CPCSEA registration number of the IAEC of CDRI is 34/1999. Colony-bred female Sprague Dawley rats were obtained from the National Laboratory Animal Centre, CSIR-CDRI. All rats were housed in a room maintained at 25°C in 12:12 hour light/dark cycles. Standard laboratory rodent chow diet and water were provided ad libitum.

Twenty four virgin adult female Sprague Dawley rats (13-15 weeks old; 220±20 g each) were randomly divided into four equal groups as follows: sham operated (ovary intact) + vehicle (gum acacia in distilled water p.o.), ovariectomized (Ovx) + vehicle, Ovx+PTH (40 µg/kg five days/week i.p.), and lactating rats (19 days post-partum, litter size of 7 pups/rat dam). For studies on estrogen deficiency-induced bone loss and restoration, twelve rats were bilaterally Ovx and left untreated for 12 weeks for osteopenia to develop, according to our previously optimized protocol [37], [38]. At this stage µCT scans (described below) revealed that all rats developed severe trabecular osteopenia (data not shown). Half the rats were given vehicle and the other half PTH for an additional 12 wk.

Rats were killed by the overdose of anesthetizing agents (ketamine, 90 mg/kg and xylazine, 10 mg/kg by i.p.). Trabecular bone at femur epiphyseal region was used for the study. Femurs were not allowed to dry at any stage of the experiments; stored at −20°C immediately after harvesting[20]. Each femur was used for the determination of BMD (g HA/cm^3^), trabecular microarchitecture (µCT) and compression testing, and the other femur for ssNMR experiments as described below. We followed previously described protocols for measuring BMD[39], micro-architecture[37], [40], [41] and compression[42], [43] parameters.

### µCT

In vivo µCT scans of femur epiphysis were obtained after 12 wk Ovx. Rats were anesthetized with ketamin (90 mg/kg) and xylazine (10 mg/kg) during the scan, which lasted about 20 min. From the in vivo scan, hundred projections were acquired at 360° angular range with special mode width adjusted to full. Reconstruction was performed using a modified Feldkamp algorithm using the SkyScan NRecon software. For parameter analysis, ROI was drawn at a total of 100 slices in the region of secondary spongiosa situated 1.5 mm away from the distal border of growth plate excluding all primary spongiosa and cortical bone, and analyzed with the CT analyzer (CTAn, SkyScan) software.

For excised femurs, the samples were scanned in batches of three at a nominal resolution (pixels) of 18 µm. Reconstruction was performed with a modified Feldkamp algorithm using the Sky Scan Nrecon software, which facilitates network distributed reconstruction to be carried out on four personal computers running simultaneously. The x-ray source was set at 70 kV and 100 mA, with a pixel size of 18 µm. A hundred projections were acquired over an angular range of 180°. The image slices were reconstructed using the cone-beam reconstruction software version 2.6 based on the Feldkamp algorithm (Skyscan). Trabecular bone was selected by drawing ellipsoid contours with the CT analyser (CTAn, Skyscan) software. Trabecular bone volume (BV/TV; percentage), trabecular number (Tb.N), and trabecular separation (Tb.Sp; millimeters) of femur epiphysis and proximal tibial metaphysis were calculated by the mean intercept length method (Gupta et al., 2009; Siddiqui et al., 2010; Verdelis et al., 2011). Trabecular thickness (Tb.Th) was calculated according to the method of Hildebrand and Ruegsegger. Connection density (Conn.D) was measured by dividing the connectivity (Conn) which is derived from Euler number, with total volume (TV) [44]. 3D parameters were based on analysis of a Marching cubes-type model with a rendered surface. CTvol software has been used to create 3D model of the bones.

Using µCT scans, BMD of femur epiphysis was determined from the VOI made for trabecular region. For calibration, the hydroxyapatite phantom rods of 2 mm of diameter with known BMD (0.25 g/cm^3^ and 0.75 g/cm^3^) were employed. For each analysis, the estimated mineral density of the bone tissue was determined based on the linear correlation between µCT attenuation coefficient and bone mineral density [45].

### Compression test

Bone mechanical strength was examined by compression test of femur head according to previously published protocols [46]. Briefly, specimens were mounted between the faces of a compression jig and a constant force was applied along the principal stress direction of the bone, i.e. in the direction of the cylinder height at a nominal deformation rate of 2 mm/min [55] using a Bone Strength Tester TK-252C (Muromachi Kikai Co., Ltd., Tokyo, Japan). The load displacement curves generated were used to calculate energy to failure (millijoule) and stiffness (Newton/mm).

### Sample preparation for ssNMR

Femur epiphysis (trabecular part) was used for NMR spectroscopy. Bone marrow was completely removed using cold saline prior to ssNMR experiment. Intact bone sample was used as it has been reported earlier that grounding disrupts internal structure [17].

### NMR spectroscopy

All NMR spectra were recorded on 600 MHz NMR spectrometer (Avance III, BrukerBiospin, Switzerland) operating at 600.154 MHz for ^1^H, 242.94 MHz for ^31^P, and 150.154 MHz for ^13^C frequencies with Bruker 3.2 mm DVT probe. Magic Angle Spinning (MAS) frequency was 10.0 kHz for all experiments. The spinning speed was controlled by Bruker MAS pneumatic unit within accuracy of ± 2 Hz. All NMR experiments were performed at 25°C. The ^1^H π/2 pulse for one pulse ^1^H NMR experiment was 2.4 µs. It was recorded with 1 k data point, acquisition time of 10.2 ms for each bone sample. For the relative change in water content, 1.4 ppm OH peak and 4.7 ppm water peak were taken for integration. Due to variation in 1H line width at 4.7 ppm water peak, we have taken area between 4 to 8 ppm for integration. Bias and slope have been corrected properly prior to integration. For ^13^C Cross Polarization (CP) spectra were recorded with ramp cross polarization sequence, 1.0 ms contact time and Small Phase Incremental Alteration (SPINAL-64) decoupling (100 kHz ^1^H r.f. field). We recorded total 6 k transients with acquisition time of 7.75 ms. For ^1^H−^31^P Heteronuclear Correlation (HetCor) experiments, the contact time was 1.0 ms and the maximum t_1_ evolution time was 1.6 ms. The effective field during ^1^H homonuclear decoupling period Phase Modulated Lee – Goldburg (PMLG)[47] were 80 kHz and high power ^1^H decoupling (100 kHz) was applied during t_2_ period. A total of 8 transients per increment and recycle delay of 4 seconds. The spectra were zero filled, and sine bell apodization was used in both dimensions prior to Fourier transformation. To determine the relative OH content, rectangular method of integration was used in each HeTCor experiment. For measurement of T_1_ relaxation time of ^31^P, inversion recovery was used with relaxation delay of 1000 seconds. Pulse length for the Rotation Echo Dipolar Recoupling (REDOR)[48] experiment were 2.5 µs for ^1^H π/2 pulse, 9 µs for ^31^P π pulse and 10 µs for ^13^C π pulse. Each REDOR experiments were recorded with de-phasing time of 16 ms with recycle delay of 3 seconds and acquisition time 6.6 ms. Because the REDOR experiment required large signal averaging due to low natural abundance (1.1%) of ^13^C in bone[9], [18], so this particular experiment included one trabecular bone sample from each group. For other NMR experiments sample size of femur epiphysis was six/group. XY-8 phase cycling on observed and dephasing channel was used to compensate pulse imperfections. The sample and probe stability for such long signal averaging was checked before experiment. For long signal averaging experiments, small sets of REDOR experiments [with (S) and without dephasing pulses (S_0_)] with 2024 scans were recorded and was added later for better signal to noise ratio.

### Statistical analysis

Data are expressed as mean ± SEM unless otherwise indicated. The data obtained in experiments with multiple treatments were subjected to one-way ANOVA followed by post hoc Newman- Keuls multiple comparison test of significance using GraphPad Prism 3.02 software. We applied leave-one-out cross validation method, and prediction accuracy was quantified by calculating the squared sample correlation coefficient (*r* ^2^).

## Supporting Information

Figure S1 ^1^H MAS NMR spectra of femur epiphysis of various groups of rat.
(JPG)
