# Supplementary figures and images for: Total Water, Phosphorus Relaxation and Inter-Atomic Organic to Inorganic Interface Are New Determinants of Trabecular Bone Integrity
Source: PLoS One. 2013 Dec 30;8(12):e83478. doi: 10.1371/journal.pone.0083478 (PMC3875436; doi:10.1371/journal.pone.0083478)

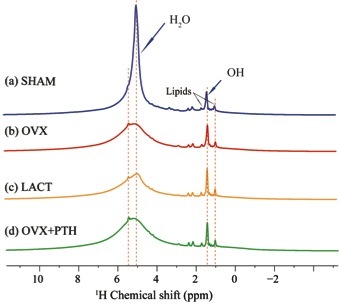

Supplement: Figure S1 — 1H MAS NMR spectra of femur epiphysis of various groups of rat. (JPG) [file pone.0083478.s001.jpg]
